# Gender differences in cognitive performance among young adults with first-episode schizophrenia in China^☆^

**DOI:** 10.1016/j.scog.2025.100353

**Published:** 2025-02-15

**Authors:** NingJing Sang, YiMin Fan, HaiYing Chen, HuiRu Cui, YanYan Wei, XiaoChen Tang, LiHua Xu, Yi Mei, JiJun Wang, TianHong Zhang

**Affiliations:** a Shanghai Mental Health Center, Shanghai Jiaotong University School of Medicine, Shanghai Engineering Research Center of Intelligent Psychological Evaluation and Intervention, Shanghai Key Laboratory of Psychotic Disorders, Shanghai 200030, PR China; b Center for Excellence in Brain Science and Intelligence Technology (CEBSIT), Chinese Academy of Science, Shanghai, PR China; c Institute of Psychology and Behavioral Science, Shanghai Jiao Tong University, Shanghai, PR China

**Keywords:** First-episode schizophrenia, MCCB, Gender difference, Cognitive performance

## Abstract

**Background:**

Individuals with schizophrenia exhibit distinctive patterns of cognitive impairments, which pose difficulties in patients' everyday functionality and reduce patients' quality of life. Previous research suggests that many demographic variables, such as gender and age, influence the cognitive performance profiles of schizophrenia patients; however, the gender differences in neurocognitive dysfunction among first-episode schizophrenia (FES) patients remain less clear.

**Methods:**

In this cross-sectional study, we compared the cognitive performance of FES patients to that of healthy controls (HC), with a specific focus on gender differences within the Chinese population aged under 35 years. Cognitive performance was assessed using the raw scores from the MATRICS Consensus Cognitive Battery (MCCB).

**Results:**

FES patients show lower overall cognitive impairment across all MCCB domains compared to HCs. Significant sex effects were observed: females outperformed males in aspects of speed of processing and verbal learning in FES, while males outperformed females in parts of working memory and reasoning and problem solving among HC patients. In both FES and HC groups, females exceeded males in visual learning. Moreover, employing a three-way multivariate analysis of variance (MANOVA) displayed interaction effects between gender and clinical diagnosis in areas of speed of processing and verbal learning.

**Conclusions:**

This suggests that schizophrenia and biological sex may jointly influence performance in these domains, emphasizing the need for early intervention and gender-sensitive approaches to address cognitive deficits in schizophrenia.

## 1 Introduction

As of 2022, the World Health Organization (WHO) reported that 24 million people worldwide suffer from schizophrenia (WHO, 2022). Individuals with schizophrenia are 2 to 3 times more likely to die prematurely compared to the general population (Laursen et al., 2014). In China, around 7 million people are affected by schizophrenia, which has a lifetime prevalence rate of 0.6 % (Yu and Lu, 2023). Nevertheless, early treatment has been shown to result in better clinical outcomes (Mesholam-Gately et al., 2009). This study aims to evaluate the nature and severity of neurocognitive dysfunction, particularly during the first episode of schizophrenia (FES), using the MATRICS Consensus Cognitive Battery (MCCB).

It is well-established that individuals with schizophrenia generally demonstrate lower cognitive performance compared to healthy controls. The illness's negative and positive symptoms often lead to cognitive decline, substantially affecting daily functional abilities (Green, 1996; Mesholam-Gately et al., 2009). Schizophrenia as a neurodevelopmental disease, involves cognitive impairment as a primary manifestation of its illness onset. Since 1988, numerous large-scale studies and meta-analysis have suggested that there have been age differences in the onset of schizophrenia between male and female patients (Angermeyer and Kühnz, 1988; Eranti et al., 2013). Therefore, it could be inferred that male and female FES patients might have different cognitive performance. Many studies have identified gender-related cognitive deficits among chronic schizophrenia (Danaher et al., 2018; Hui et al., 2016; Rodriguez-Jimenez et al., 2015; Nitzburg et al., 2014; Zhang et al., 2017). For instance, males with chronic schizophrenia in Chinese population demonstrated inferior performance, such as in social cognition, processing speed, verbal, and visual learning compared to females (Zhang et al., 2017). Given that healthy males generally performed better in these cognitive domains, the worsened scores of males with schizophrenia compared to females might indicate that males experience a greater cognitive decline after diagnosing schizophrenia. However, gender differences in the early stages of diagnosis are less investigated in the current field. This indicates a need to assess FES to explore whether the specific gender patterns of cognitive decline are also evident in participants who have recently experienced the onset of full-stage schizophrenia.

The consensus on the typical age of onset for schizophrenia is between 20 and 30 years (Schimmelmann et al., 2007; O'Donoghue et al., 2015), with males have an earlier onset by 1.49 years (Eranti et al., 2013). Beyond the limited findings, research on sex differences in cognitive performance in FES remains inconsistent. For instance, Danaher et al. (2018) reported gender differences on verbal comprehension that male FES outperformed female counterparts. Whereas, Pu et al. (2019) reported the converse findings that FES female exceeded male on verbal learning and memory tasks. The discrepancies between studies may stem from varied age ranges, capturing different stages of psychotic symptom onset (Danaher et al., 2018), and a failure to control for treatment or other demographic differences, and absence of control group for comparison. Our study aims to fill this gap by focusing on FES adults age between 20 and early 30 years old and compared with a control group, specifically within the Chinese population. We employed raw scores to enhance generalizability and test-retest reliability, and to facilitate direct outcome comparisons without the influence of population adjustments, such as those used T-scores. Our research aims include:

1. Investigate sex differences in neurocognition among FES individuals, and compare the patterns with a control group.
2. Examine the relationship and interaction effects between gender, clinical conditions, and cognitive performance, using age and education as covariates.

## 2 Method

### 2.1 Study design and participants

This cross-sectional study utilized data from China's National Key R&D Program (2016YFC1306800), led by the Shanghai Mental Health Center (SMHC) and collected from 2016 to 2021. Ethical approval was granted by the SMHC's Research Ethics Committee (IRB2016-009), and data collection was conducted across ten psychiatric hospitals in China, focusing on identifying biological markers for psychosis and developing early intervention treatments. Ethical clearance was obtained from all sites, with informed consent from participants, adhering to the 1975 Declaration of Helsinki's ethical guidelines, as amended in 2008.

The study included 660 subjects age over 21, with 273 FES patients and 387 healthy controls (HC) adults. FES diagnoses followed the DSM-IV-TR criteria, and schizophrenia severity was assessed with the Positive and Negative Syndrome Scale (PANSS) (Kay et al., 1987). Exclusion criteria included a history of substance abuse for all and any prior mental health issues for controls.

### 2.2 Neurocognitive assessment

The MCCB, developed in 2004 by Green et al., standardizes cognitive function measurement for schizophrenia research, covering seven domains through ten tests. These domains are: 1) Speed of processing (Brief Assessment of Cognition in Schizophrenia (BACS): Symbol-Coding, Category Fluency: Animal Naming, Trail Making Test: Part A), 2) Attention/vigilance (Continuous Performance Test—Identical Pairs), 3) Working memory (Wechsler Memory Scale®—3rd Ed and Letter-Number Span), 4) Verbal learning (Hopkins Verbal Learning Test—Revised), 5) Visual learning (Brief Visuospatial Memory Test-Revised), 6) Reasoning and problem solving (Neuropsychological Assessment Battery's Mazes), and 7) Social cognition (Mayer-Salovey-Caruso Emotional Intelligence Test). As translated to simplified Chinese, MCCB shows high clinical validity and reliability in schizophrenia and healthy controls (Shi et al., 2015; Zhang et al., 2019). The Letter-Number Span test was excluded due to linguistic differences (Zhang et al., 2019) and the Social Cognition was excluded as it automatically computed adjusted scores of genders and age, which did not fit into the nature of our study.

### 2.3 Demographics

Biological sex, age, educational background, and parental education were documented. The lowest age was 22 and the highest was 33 to capture the peak onset of schizophrenia during early adulthood.

### 2.4 Clinical assessment

The diagnosis of FES adhered to the Diagnostic and Statistical Manual of Mental Disorders, Fourth Edition, Text Revision (DSM-IV-TR). The PANSS was employed to evaluate clinical symptoms encompassing positive, negative, and general psychopathology, utilizing a 7-point Likert scale. Structured interviews were conducted by psychiatrists who had received training specifically for this research. Among these trained interviewers, the inter-rater reliability score for the PANSS assessment reached 0.92. (Zhang et al., 2023).

## 3 Statistical analysis

Data analysis utilized IBM® SPSS® Statistics Version 29.0. Gender and group differences (FES and HC) on demographic variables were analyzed using *t*-tests and chi-square tests. The raw scores of neurocognitive data were analyzed using a three-way multivariate analysis of variance (MANOVA), assessing the impact of groups and sex on cognitive performance across eight MCCB domains, controlling age and education as covariates. This analysis identified main effects and interactions of clinical condition and sex into binary formats. Outliers were excluded. Effect sizes were defined as *η*^*2*^ = 0.01 (small), *η*^*2*^ = 0.06 (medium), and *η*^*2*^ = 0.14 (large). When the multivariate effect was significant at the *p* < 0.05 level, stepdown analyses were performed to evaluate specific interactions among four groups divided by sex and clinical conditions.

## 4 Results

A total of 660 valid responses were analyzed (Table 1), with 305 males and 355 females participating. Of these, 41.4 % (*N* = 273) experienced their first episode of schizophrenia, while 58.6 % (*N* = 384) were healthy controls. There were 171 males and 216 females in HC group, and 134 male and 139 females in FES group. The participants' mean age was 25.3 years, and the minimum age was 22 and maximum was 33. The average education was 14.58 years, indicating some college or higher education post-high school. Gender difference was observed in HC group, where females have higher education than males (*t* (385) = −2.25, *p* = 0.02). Other than age in FES, no other gender differences were reflected from the results.

**Table 1:** Clinical demographics: education, parental education, and age mean scores and gender differences in groups of healthy control (HC), and first episode schizophrenia (FES).

Variables	First episode schizophrenia (FES)				Healthy control (HC)			
	Male [M (SD)]	Female [M (SD)]	*t*	*p*	Male [M (SD)]	Female [M (SD)]	*t*	*p*
Education [Years]	11.81 (3.29)	12.71 (3.35)	−2.54	0.25	15.91 (2.40)	16.44 (2.20)	−2.25	0.02
Father's Education [Years]	9.43 (3.78)	10.20 (3.31)	−1.20	0.23	10.91 (3.04)	10.81 (3.50)	0.28	0.77
Mother's Education [Years]	8.68 (4.29)	8.95 (3.78)	−0.36	0.72	10.06 (3.42)	9.95 (3.63)	0.27	0.78
Age [Years]	25.54 (2.41)	26.15 (2.55)	−2.04	0.04	24.95 (2.25)	25.00 (2.39)	−0.17	0.86

Note: M = mean; SD = standard deviation; *t* = t scores of independent sample t-test.

Among FES (Table 2), females excelled over males in BASC (*t* (271) = −3.39, *p* = 0.001), HVLT (*t* (271) = −3.05, *p* = 0.003), and BVMT (*t* (271) = −2.44, *p* = 0.02). However, in HC, female only outperformed male on BVMT (*t* (385) = −1.99, *p* = 0.04). Although gender differences on WMS-III and NAB were small, male outperformed female on these two tests (WMS-III: *t* (385) = 3.13, *p* = 0.002; NAB: *t* (385) = 2.96 *p* = 0.003). PANSS scores were compared for FES group, but no significant gender difference was found (Table 3). HC outperformed FES in all eight cognitive domains.

**Table 2:** Gender differences in cognitive abilities (mean, SD, t, and *p* values) among first episode schizophrenia (FES) patients and healthy controls (HC).

Variables	Mean (SD)		Comparisons	
FES	Male	Female	*t*	*p*
TMT-A	54.03 (36.10)	49.4(25.97)	1.221	0.22
BACS:SC	42.15 (12.16)	47.15(12.23)	−3.387	<0.001
HVLT	19.07 (6.13)	21.27(5.78)	−3.051	<0.001
WMS-III: SS	13.98 (3.53)	13.81(3.60)	0.398	0.70
NAB	12.10 (7.06)	10.53(6.98)	1.842	0.07
BVMT	18.71 (8.12)	21.14(8.29)	−2.443	0.02
CF	17.81 (5.84)	18.05(5.56)	−0.354	0.72
CPT-IP	1.87 (0.88)	1.76(0.81)	1.138	0.26

HC	Male	Female	*t*	*p*
TMT-A	27.75 (8.84)	28.86 (10.08)	−1.13	0.26
BACS:SC	64.35 (10.82)	65.23 (9.73)	−0.84	0.40
HVLT	26.27 (4.06)	26.65 (4.40)	−0.86	0.39
WMS-III: SS	16.99 (2.92)	16.04 (3.01)	3.13	0.002
NAB	19.65 (4.77)	18.16 (5.04)	2.96	0.003
BVMT	27.67 (5.45)	28.73 (5.00)	−1.99	0.04
CF	24.01 (5.99)	24.23 (5.25)	−0.39	0.07
CPT-IP	3.00 (0.61)	3.04 (0.56)	−0.60	0.54

Total	FES	HC	*t*	*p*
TMT-A	51.67 (31.38)	28.37 (9.56)	−11.88	<0.001
BACS:SC	44.70 (12.43)	64.84 (10.22)	22.02	<0.001
HVLT	20.19 (6.04)	26.48 (4.25)	14.82	<0.001
WMS-III: SS	13.89 (3.56)	16.46 (3.01)	9.72	<0.001
NAB	11.30 (7.05)	18.82 (4.97)	15.13	<0.001
BVMT	19.95 (8.28)	28.26 (5.22)	14.65	<0.001
CF	17.93 (5.69)	24.13 (5.59)	13.89	<0.001
CPT-IP	1.81 (0.85)	3.02 (0.58)	20.43	<0.001

Note: TMT: Trail Making Test: Part A; BACS:SC: Brief Assessment of Cognition in Schizophrenia (BACS): Symbol-Coding; HVLT: Hopkins Verbal Learning Test- Revised; WMS-III:SS: Wechsler Memory Scale—3rd Ed.(WMS-III): Spatial Span; NAB: Neuropsychological Assessment Battery: Mazes; BVMT-R: Brief Visuospatial Memory Test- Revised; CF: Category Fluency: Animal Naming; CPT-IP: Continuous performance test- Identical Pair.

**Table 3:** Gender differences in PANSS scores among First Episode Schizophrenia (FES) Patients.

FES	Male	Female	*t*	*p*	Total
	Mean (SD)				Mean (SD)
PANSS_P	22.05 (5.92)	22.55(5.78)	−0.708	0.479	22.31 (5.84)
PANSS_N	18.25 (7.29)	18.60(6.60)	−0.426	0.671	18.43 (6.93)
PANSS_G	39.85 (8.52)	39.62(8.9)	0.220	0.826	39.73 (8.70)
PANSS_Total	80.15 (17.22)	80.78(17.1)	−0.302	0.763	80.47 (17.13)

Note: PANSS_P: PANSS Positive; PANSS_N: PANSS Negative; PANSS_G: PANSS General.

After controlling age and education, the MANOVA tests (Table 4) showed that diagnosis had the strongest impact on cognitive performance across all tests (*p* < 0.001) with Pillai's Trace = 0.31, *p* < 0.001, and partial *η*^*2*^ = 0.31, *p* = 0.002, and partial *η*^*2*^ = 0.31. Interaction effects of gender and diagnosis were significant for BASC and HVLT-R with Pillai's Trace = 0.037, *p* = 0.002, and partial *η*^*2*^ = 0.037. Gender had significant effect on MCCB performance on BACS:SC, HVLT-R, WMS-III, NAB, and BVMT-R with Pillai's Trace = 0.10, *p* < 0.001, and partial *η*^*2*^ = 0.10).

**Table 4:** MANOVA results for each cognitive domain including main and interaction effects.

Cognitive domain	Effect	*F*	*p*	Partial *η*^*2*^
TMT:Part A	Gender	0.05	0.82	<0.001
	Diagnosis	53.39	<0.001	0.076
	Gender × Diagnosis	2.47	2.47	0.004
BACS: SC	Gender	6.35	0.01	0.010
	Diagnosis	206.38	<0.001	0.240
	Gender × Diagnosis	5.23	5.23	0.008
HVLT-R	Gender	5.89	0.02	0.009
	Diagnosis	73.02	<0.001	0.101
	Gender × Diagnosis	4.86	4.86	0.007
WMS-III: SS	Gender	7.64	0.006	0.010
	Diagnosis	28.66	<0.001	0.042
	Gender × Diagnosis	2.12	0.15	0.003
NAB:Mazes	Gender	19.74	<0.001	0.030
	Diagnosis	79.48	<0.001	0.109
	Gender × Diagnosis	0.11	0.74	<0.001
BVMT-R	Gender	6.42	0.01	0.010
	Diagnosis	85.25	<0.001	0.115
	Gender × Diagnosis	1.22	0.27	0.002
CF: AN	Gender	0.08	0.77	<0.001
	Diagnosis	57.75	<0.001	0.081
	Gender × Diagnosis	0.03	0.87	<0.001
CPT-IP	Gender	3.09	0.08	0.005
	Diagnosis	180.23	<0.001	0.216
	Gender × Diagnosis	2.74	0.10	0.004

Note: Age and Education were controlled as covariates. TMT: Trail Making Test: Part A; BACS:SC: Brief Assessment of Cognition in Schizophrenia (BACS): Symbol-Coding; HVLT: Hopkins Verbal Learning Test- Revised; WMS-III:SS: Wechsler Memory Scale—3rd Ed.(WMS-III): Spatial Span; NAB: Neuropsychological Assessment Battery: Mazes; BVMT-R: Brief Visuospatial Memory Test- Revised; CF:AN: Category Fluency: Animal Naming; CPT-IP: Continuous performance test- Identical Pair.

**Fig. 1:**
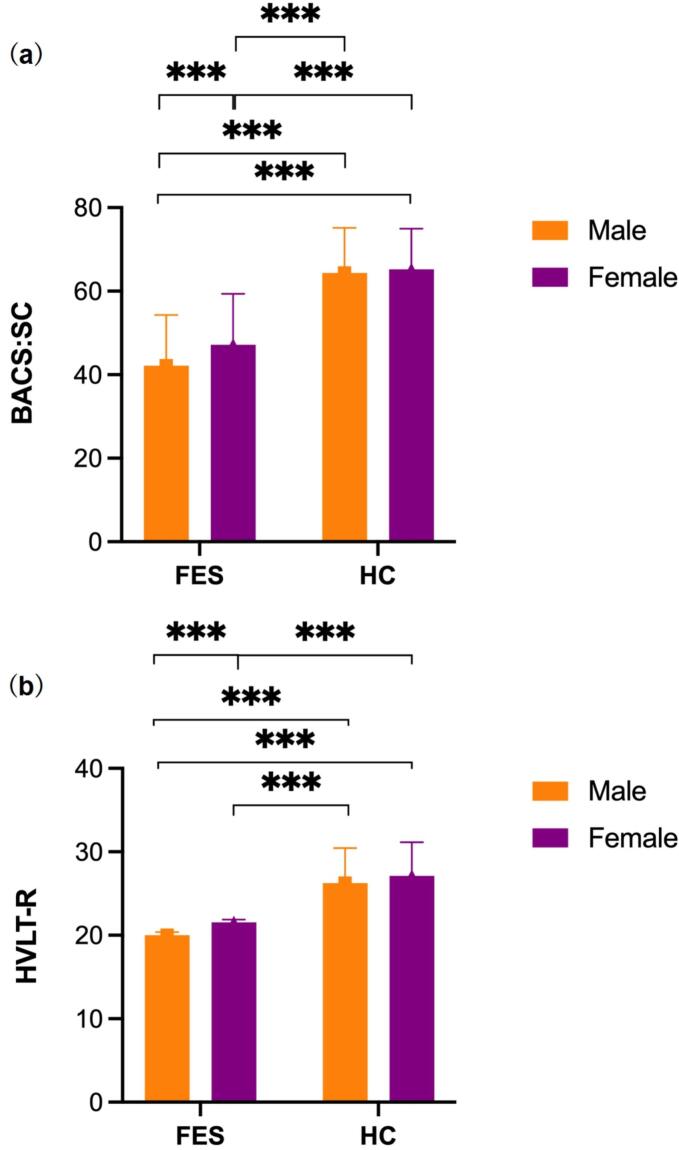
Comparing of the mean BACS:SC scores (1a) and HVLT-R scores (1b) among FES females, FES males, HC females, and HC males.

Post-hoc analysis (Fig. 1) of MANOVA results further demonstrated gender difference pattern between FES and HC. For BACS mean scores (Fig. 1a), *F* (3, 656) = 181.03, *p* < 0.001, Mean scores were: Mean for HC male = 64.35, SD = 10.82; Mean for FES male = 42.15, SD = 12.16; Mean for HC female = 65.23 SD = 9.73; Mean for FES female = 47.15, SD = 12.24. For HVLT-R scores (Fig. 1b), *F* (3,656) = 88.32. Mean scores were: Mean for HC male = 26.27, SD = 4.06; Mean for FES male = 19.07, SD = 6.13; Mean for HC female = 26.65, SD = 4.39; Mean for FES female = 21.27, SD = 5.78.

## 5 Discussion

Our findings on FES and HC supported the presence of broad cognitive impairment in people who experienced their first full episode of schizophrenia. Cognitive impairments presented in both overall and specific cognitive domains. We also clarified sex differences in neurocognition among FES adults in their early adulthood—a less explored area in this field. Key findings include: (1) HC participants outperformed FES participants in all eight MCCB cognitive domains (2) Significant sex effects were observed, with females outperforming males on BACS, HVLT-R, and BVMT in FES, and male outperformed female in WMS-III and NAB among HC patients. For both FES and HC, female outperformed male on BVMT; (3) A significant interaction between sex and clinical diagnosis were noted for BACS and HVLT-R; (4) A post-hoc analysis revealed significant inter-group comparison of gender between FES and HC.

The significant differences in all eight MCCB domains between FES and HC were consistent and robust with existing literature (Cai et al., 2024; Zhao et al., 2021). The impairments suggested a global cognitive deficit for participants after experiencing a full episode of schizophrenia. Notably, in our results, the BACS domain exhibited the largest t-score, indicating a more substantial effect size that clinical symptoms of schizophrenia might have the largest effect on BACS task. This finding was also identified in a recent meta-analysis on Chinese FES population, where the BACS raw score demonstrated the greatest Standard Mean Differences (Zhang et al., 2019).

In our study, females with schizophrenia were older on average and outperformed males in three out of seven cognitive tests (i.e. BACS, HVLT-R, BVMT-R). BACS was the strongest predictor in assessing speed of processing (Burton et al., 2013); HVLT-R tested participants' verbal learning and memory (Brandt, 1991); BVMT-R assessed visual learning and memory. Performance on these tests might be linked. Tam and Schmitter-Edgecombe (2013) suggested that BACS test scores strongly predicted processing speed, and speed of processing could predict the performance on BVMT-R. Hence, in our results, BACS deficits in men might also relate to their lower BVMT-R scores. However, it should be noted that we did not conduct correlation analyses on the MCCB variables to explore this potential connection. In terms of verbal and visual learning impairments in men with FES, as reflected by HVLT-R and BVMT-R scores, impairment profiles aligned with seen in chronic schizophrenia (Zhang et al., 2017). This might suggest the impairments on these two domains were more sustaining through the stage of schizophrenia for men. Female's better performance might result from the protective role of estrogen on cognitive function, which can modulate dopamine D2-receptors and elicit an antipsychotic effect (Häfner, 2003). Together with progesterone, both sexual hormones elicited neuroprotective properties and restored female cognitive ability from schizophrenia symptoms (McGregor et al., 2017).

Female superiority on HVLT-R aligned with Ryan et al. (2020), who found that being female and had younger age were significantly associated with better recall on HVLT-R test. This advantage persisted even after adjusting for disease severity (Pu et al., 2019). The researchers concluded that gender differences may suggest neurocognitive functioning as a ‘trait index’, unaffected by disease phase. However, female also showed superior performance in traditionally male-dominated domains, like visual learning (i.e. BVMT-R). Interestingly, our post-hoc comparisons suggested that although BACS and HVLT-R performance indicated cross-group differences, no gender differences were found in control. This finding supported Zhang et al. (2017)’s explanation that men might have more impairment on cognition compared to female after experiencing the full episode of schizophrenia, particularly in verbal ability, because those similar patterns of gender differences had not been observed in healthy controls. Stress might be another explanation. Previous study based on Cortisol Awakening Response (CAR) indicated that, high stress was associated with poorer cognition in verbal memory and processing speed in individuals with FES (Aas et al., 2011). In our study, when FES participants were assessed in clinical setting, they might be more nervous compared to healthy control, therefore, had worse outcomes than control. Taken the protective role of estrogen in females, together stress and sex hormones might explain these post-hoc results.

When taking gender and clinical condition together, gender and clinical condition displayed significant interactional effect on BACS and HVLT-R. This was consistent with Zhao et al. (2021), who reported similar interactions on BACS and HVLT-R. However, while they also found effects in four other domains, they did not report effect sizes in their data which complicated direct comparison with our findings. In addition, they compared drug-naïve FES with healthy control, but in our study, we did not account for medical interventions, highlighting the need for future research to evaluate medication's effects on neurocognition among FES population.

Nevertheless, cognitive impairments could be improved. A longitudinal study showed that FES patients might be able to improve their speed of processing and visual learning through better control of their clinical symptoms, and even simple progression of time could substantially restore cognitive performance, though their improvement are still not comparable with control (Mohn and Torgalsbøen, 2018). These insights highlight the potential for interventions such as cognitive therapy or advanced pharmacological treatments to enhance cognitive performance in schizophrenia patients. For instance, a case-control study demonstrated that low-dose antipsychotic treatment could improve performance across all MCCB domains within six months, though no further improvements were observed at the 12-month mark (Olivier et al., 2015). This underscored the importance of cognitive restoration in treatment strategies and the need to understand the relationship between PANSS symptoms and cognitive function. Furthermore, as brain stimulation therapies like Transcranial Magnetic Stimulation (TMS) become increasingly accessible, our findings offered valuable guidance for clinicians in customizing treatments for FES patients, considering the gender differences in neurocognition we observed.

### 5.1 Limitation

Our study did not adjust for psychotic symptom severity or antipsychotic medication use, known to influence overall cognitive performance and particular attention-vigilance (Cai et al., 2024). Additionally, we didn't account for psychosis onset age, grouping participants by current conventions and sample size without considering psychosis duration or onset. Future studies would benefit from adjusting those confounders.

Furthermore, many statistical distributions in the dataset violated the assumptions of normality and homogeneity of variances. However, according to the Central Limit Theorem (Dudley, 1978), the sampling distribution of the mean tended to normally distribute when having a sufficiently large sample size. Moreover, although variance differences between groups in large samples might appear unequal in variance tests like Levene's test, these might not be substantial enough to invalidate parametric tests. Field (2009) suggested that a sample size >200 could be considered large. Therefore, given the large sample size (*N* = 660) in this study, most analyses proceeded under the assumptions of normal distribution and equal variances. Furthermore, given the scarcity studies suing MCCB raw scores, some of our findings are compared with T-score studies. Although two types of results based on different calculation, their suggested tendency are transferable. To reduce confusion, all comparisons with T scores have been explicitly noted and clarified for reader.

Finally, the study had the drawback of all cross-sectional studies that limited causal inferences. Nevertheless, our research team had minimized the drawback through meticulous medical setting assessments which carried out by trained clinicians. Our study, unlike many with small samples, had a sufficient size for meaningful results.

## 6 Conclusion

This study elucidated the significant cognitive impairments faced by individuals with the first episode of schizophrenia, with a notable focus on gender differences in cognitive performance. It revealed that FES patients experience widespread cognitive deficits from the onset of the illness, with females generally showing better cognitive outcomes than males. These findings highlighted the need for gender-specific approaches in the treatment and management of schizophrenia, emphasizing the importance of addressing cognitive impairments early in the disease to improve patient outcomes.

## CRediT authorship contribution statement

**NingJing Sang:** Writing – review & editing, Writing – original draft, Visualization, Software, Methodology, Investigation, Formal analysis, Data curation. **YiMin Fan:** Conceptualization, Investigation, Methodology, Writing – review & editing. **HaiYing Chen:** Conceptualization, Investigation, Methodology, Writing – review & editing. **HuiRu Cui:** Resources. **YanYan Wei:** Resources, Writing – review & editing. **XiaoChen Tang:** Resources, Project administration. **LiHua Xu:** Software, Resources. **Yi Mei:** Resources. **JiJun Wang:** Supervision, Resources, Funding acquisition. **TianHong Zhang:** Writing – review & editing, Supervision, Resources, Project administration, Funding acquisition, Conceptualization.

## Funding

This study was supported by the 10.13039/100007225Ministry of Science and Technology of China, 10.13039/501100012166National Key Research and Development Program of China (2023YFC2506800), 10.13039/501100001809National Natural Science Foundation of China (82171544, 82371505, 82151314, 82101623).

## Declaration of competing interest

None declared.
